# The microglial NADPH oxidase complex as a source of oxidative stress in Alzheimer's disease

**DOI:** 10.1186/1742-2094-3-30

**Published:** 2006-11-09

**Authors:** Brandy L Wilkinson, Gary E Landreth

**Affiliations:** 1 Alzheimer Laboratory, Department of Neuroscience, Case Western Reserve University, Cleveland, OH 44106, USA

## Abstract

Alzheimer's disease is the most common cause of dementia in the elderly, and manifests as progressive cognitive decline and profound neuronal loss. The principal neuropathological hallmarks of Alzheimer's disease are the senile plaques and the neurofibrillary tangles. The senile plaques are surrounded by activated microglia, which are largely responsible for the proinflammatory environment within the diseased brain. Microglia are the resident innate immune cells in the brain. In response to contact with fibrillar beta-amyloid, microglia secrete a diverse array of proinflammatory molecules. Evidence suggests that oxidative stress emanating from activated microglia contribute to the neuronal loss characteristic of this disease. The source of fibrillar beta-amyloid induced reactive oxygen species is primarily the microglial nicotinamide adenine dinucleotide phosphate (NADPH) oxidase. The NADPH oxidase is a multicomponent enzyme complex that, upon activation, produces the highly reactive free radical superoxide. The cascade of intracellular signaling events leading to NADPH oxidase assembly and the subsequent release of superoxide in fibrillar beta-amyloid stimulated microglia has recently been elucidated. The induction of reactive oxygen species, as well as nitric oxide, from activated microglia can enhance the production of more potent free radicals such as peroxynitrite. The formation of peroxynitrite causes protein oxidation, lipid peroxidation and DNA damage, which ultimately lead to neuronal cell death. The elimination of beta-amyloid-induced oxidative damage through the inhibition of the NADPH oxidase represents an attractive therapeutic target for the treatment of Alzheimer's disease.

## Background

Alzheimer's disease (AD) is the most common form of senile dementia and is characterized by progressive cognitive impairment and profound neuronal loss. The neuropathological hallmarks of AD are the senile plaques consisting of extracellular deposits of fibrillar β-amyloid (fAβ) and the intracellular neurofibrillary tangles composed of hyperphosphorylated tau. While the events leading to AD onset remain elusive, the progression of disease pathology has been thoroughly examined and several mechanisms of neuronal damage have been identified. Considerable attention has been focused on the role of inflammatory mechanisms in the etiology of AD, and senile plaques are the site of local inflammatory response [1-3]. Epidemiological studies also provide persuasive evidence for the involvement of inflammatory mechanisms in AD. Patients using nonsteroidal anti-inflammatory drugs (NSAIDs) were found to have a dramatically reduced incidence of disease. These studies reported that patients treated with NSAIDs for a 2-year period or more had as much as 60–80% reduction in the risk for AD [4-6]. Long-term NSAID treatment attenuated disease onset, slowed the rate of cognitive impairment and reduced the level of overall symptomatic severity [6,7]. In both humans and mice, NSAID treatment is associated with reduced Aβ plaque burden and a reduction in plaque-associated microglia [8-11]. NSAIDs have also been shown to exhibit pleiotropic effects on the processing of the amyloid precursor protein (APP) [12-14]. These data support the potential utility of anti-inflammatory drug therapies in the treatment of AD.

Microglia are the principle immune effector cells in the brain and represent approximately 5–10% of all glia found in the brain. The density of microglia in the brain is approximately 6 × 10^3 ^cells/mm^3 ^[15]. Microglia are derived from a myeloid lineage, and originate from bone marrow-derived progenitor cells that are trafficked from the periphery into the brain parenchyma where they differentiate [16-19]. It has recently been appreciated that "resting" or "quiescent" microglia are highly dynamic and constantly extend their processes to survey their microenvironment. This surveillance permits quick reaction to either local injury or invading pathogens [15,20].

In the AD brain, microglia are characterized by a reactive phenotype and are found surrounding senile plaques [21]. These cells mount a local inflammatory response and express cell surface receptors reflective of the phenotypic activation of this cell type [22]. Specifically, activated microglia secrete inflammatory cytokines such as tumor necrosis factor alpha (TNF-α), interleukin-1 beta (IL-1β), IL-6, and chemokines, all of which are found at elevated levels in the AD brain [3]. Fibrillar Aβ-stimulated microglia also release reactive oxygen species (ROS) and reactive nitrogen intermediates (RNI) [3]. A chronic microglial inflammatory response leads to the continued release of inflammatory mediators that are associated with neurotoxic injury to surrounding neurons (Goerdt and Orfanos 1999; Wyss-Coray and Mucke 2002).

In the CNS, oxidative damage generally manifests as lipid peroxidation and the formation of protein oxidation products that are toxic to neurons. Neurons are inherently susceptible to oxidative damage because of high respiratory turnover, dependency on oxidative phosphorylation reactions, high concentrations of catalytic iron and low levels of antioxidant defense enzymes [23]. Oxidative damage is observed early in the progression of AD [24,25], and can be detected prior to fAβ deposition both in the human brain [26] and animal models of the disease [24]. These findings suggest that oxidative damage emanating from the reactive microglia and astrocytes adjacent to senile plaques may play an early role in the pathogenesis of AD.

Several potential sources of ROS exist within microglia and astrocytes including the nicotinamide adenine dinucleotide phosphate (NADPH) oxidase, mitochondria respiratory chain, xanthine oxidase, microsomal enzymes, cycloxygenase and lipoxygenase. In response to fAβ; however, it is believed that the primary source of ROS and the source of widespread oxidative damage found in both AD brains and mouse models of AD is the microglial NADPH oxidase [26-30].

Despite ample evidence supporting microglial NADPH oxidase participation in fAβ-stimulated ROS production and oxidative damage, until recently little was known about the signaling pathway(s) subserving NADPH oxidase assembly. A mechanistic linkage between Aβ fibril engagement of the cell surface receptor complex and the initiation of intracellular signaling events regulating oxidase assembly and activation has been described [31,32].

## The NADPH oxidase

The phagocytic NADPH oxidase plays an essential role in innate immunity by catalyzing the formation of superoxide (O_2_^-^), which facilitates the destruction of invading microorganisms during phagocytosis. Upon oxidase activation, O_2_^- ^is produced and participates in microbial killing. However, other more potent ROS are rapidly formed including hydrogen peroxide, hydroxyl radical, peroxynitrite and other oxidants [33]. The excessive production of these free radicals can damage tissue adjacent to the sites of inflammatory action; therefore, the activation of the NADPH oxidase is tightly controlled though regulated assembly of the individual oxidase subunits into a functionally active complex [34].

**Figure 1:**
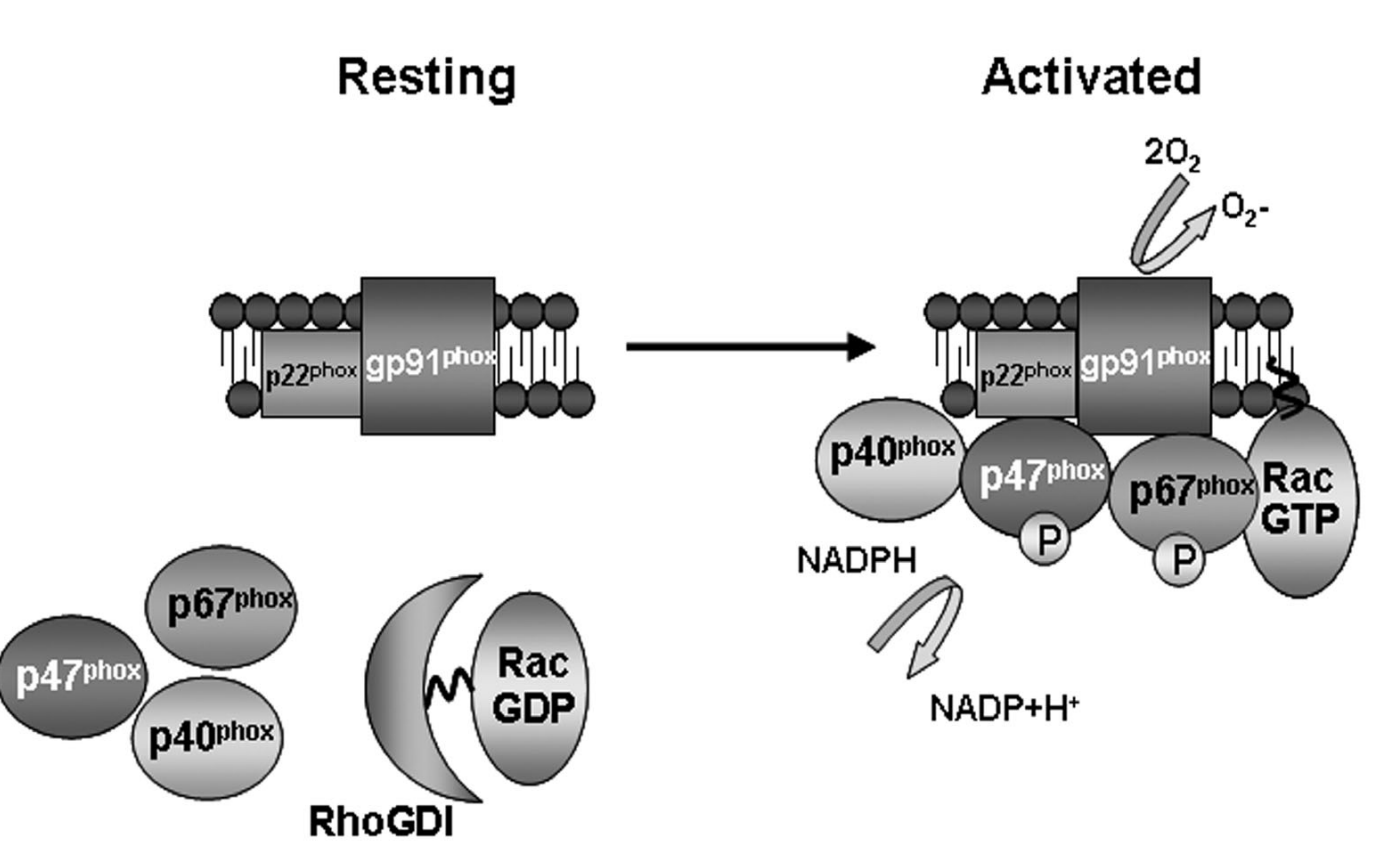
Activation of the phagocytic NADPH oxidase complex. Stimulation of the phagocyte induces the parallel activation of oxidase components within the cytoplasm. This activation causes the conversion of Rac into an active GTP-bound form and the phosphorylation of p47^phox ^and p67^phox^. These subunits then translocate to the membrane where they interact with p22^phox ^and gp91^phox ^(NOX2) to initiate reactive oxygen production.

The NADPH oxidase consists of two integral membrane proteins, p22^phox ^and gp91^phox^, which together form a heterodimeric flavoprotein known as cytochrome b_558 _(Figure 1). In addition, there are four cytosolic components p47^phox^, p67^phox^, p40^phox^, and the small G-protein Rac (Figure 1). Activation of the NADPH oxidase occurs when the macrophage/microglial cell is exposed to inflammatory stimuli initiating the activation of multiple parallel intracellular signaling cascades. These cytoplasmic signaling events stimulate the phosphorylation of p47^phox ^and p67^phox ^and the GDP/GTP exchange on Rac. The cytosolic components then translocate to the membrane where they form a complex with cytochrome b_558_. The oxidase complex then initiates electron flow and generation of O_2_^- ^through the NADPH-derived electron reduction by the flavocytochrome (Figure 1).

In recent years, it has become evident that several different forms of the NADPH oxidase exist and comprise a family of related enzymes, which are differentially expressed in a variety of tissue types. While the presence of these various subunits in different tissue types remains under debate, it is clear that ROS function more broadly than as simply one consequence of the immunological response. The defining element of these enzyme complexes is the catalytic gp91 subunit. A new nomenclature has now been applied to this family of enzymes with classification based on the structure of the gp91 subunit. The classical phagocytic NADPH oxidase incorporates the gp91 subunit and is termed NOX2 [35].

Cytosolic p47^phox ^and p67^phox ^are often found as a complex and both require phosphorylation prior to their translocation to the membrane [34,36]. Analysis of p47^phox ^and p67^phox ^phosphorylation has been investigated with the use of specific protein kinase inhibitors revealing that protein kinase C (PKC) [37-39], p21-activated kinase-1 (PAK1) [40], mitogen-activated protein kinase (MAPK) [41], Akt [42], and phosphatidylinositol-3 kinase (PI3K) [43] phophorylate p47^phox ^or p67^phox^. The diversity of kinases implicated in the phosphorylation of p47^phox ^and p67^phox ^suggests that the intracellular signaling pathways responsible for this phosphorylation event are complex and may be cell type and stimulus specific.

**Figure 2:**
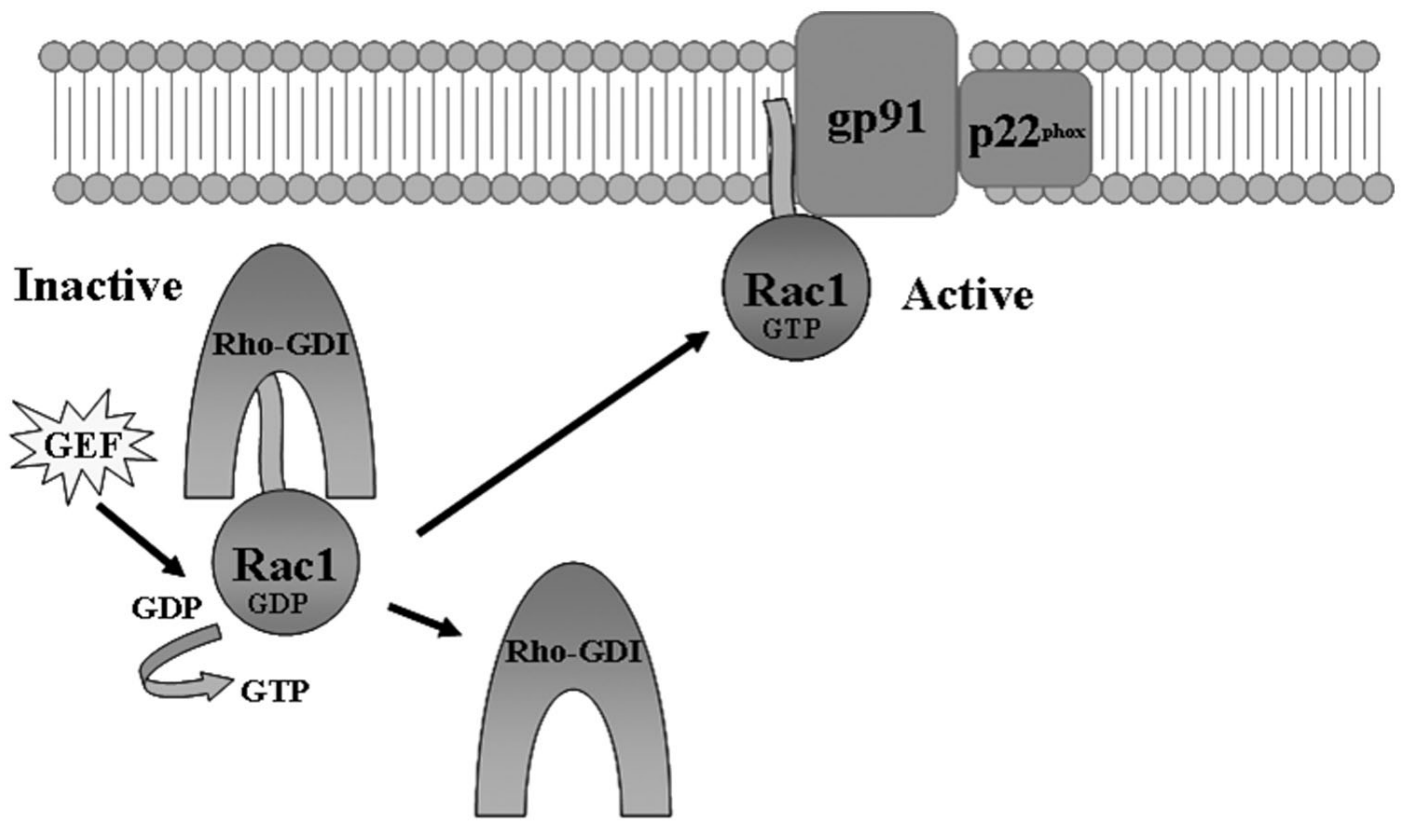
Activation of the Rac GTPase. GTP-binding of the Rac protein confers an "active" confirmation allowing Rac to modulate downstream effectors. Rac is regulated by upstream effectors know as GEFs which facilitate the exchange of GDP for GTP and increase the amount of active Rac.

There are several isoforms of the Rac GTPase, with Rac1 being the predominant isoform in macrophages and monocytic cells while Rac2 is predominantly expressed in neutrophils [44]. Rac is a member of the Rho-family of small monomeric GTPases. Like all Ras-superfamily members, Rac1 acts as a molecular switch cycling between an inactive guanosine diphosphate (GDP)-bound state and an active guanosine triphosphate (GTP)-bound state that can bind target proteins (Figure 2). This process is facilitated by a group of molecules known as guanine-nucleotide exchange factors (GEFs). In resting cells, Rac is anchored in the cytosol through an interaction between its C-terminal prenyl moiety and the GDP dissociation inhibitor, RhoGDI. During activation by an inflammatory stimulus, Rac binds GTP and dissociates from RhoGDI. Rac is then able to translocate to the plasma membrane, where its prenyl group inserts into the membrane, tethering Rac to its inner face and facilitating Rac's interaction with p67^phox ^[45]. While the precise role of Rac in NADPH oxidase activation is not fully understood, several lines of evidence suggest Rac acts as an adaptor molecule for p67^phox ^[46,47], and may be actively involved in the electron transfer process itself [48,49]. The requirement for Rac-GTP has been further established through *in vitro *studies demonstrating that the addition of dominant-negative Rac or overexpression of Rho-GDI severely diminishes superoxide production [50,51].

## Microglia and the NADPH oxidase

**Figure 3:**
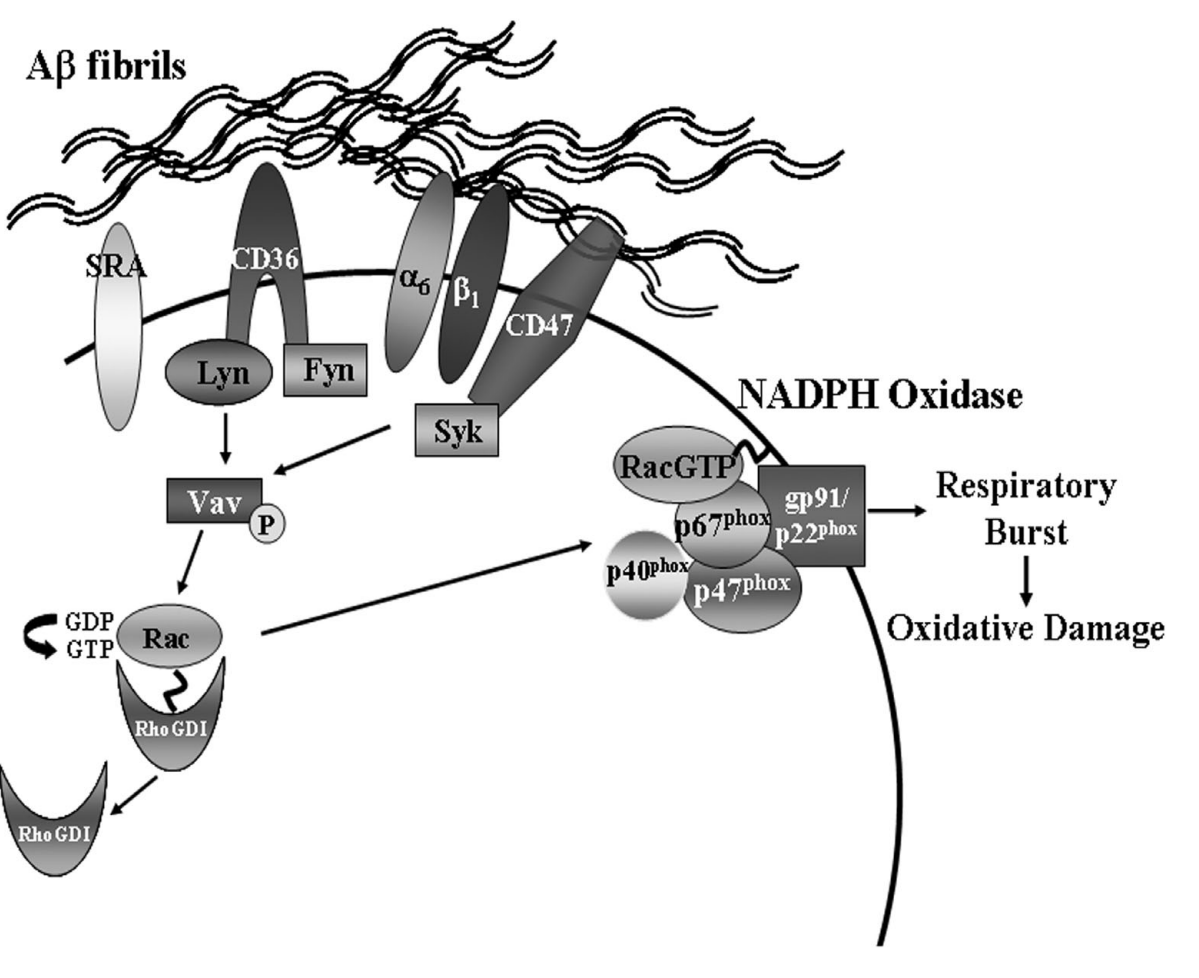
Model of intracellular signaling following Aβ fibril interaction with the microglial cell surface receptor complex. Fibrillar engagement of an ensemble of cell surface receptors initiates a tyrosine kinase-based signaling cascade. Tyrosine phosphorylation of the Vav-GEF results in the activation of downstream Rac1 GTPase.

We have recently described a multireceptor cell surface complex for fAβ on microglia [32]. Microglial contact with fAβ catalyzes the assembly of an ensemble of cell surface receptors that includes CD36, α_6_β_1 _integrin, CD47, and the class A scavenger receptor (SRA) (Figure 3). Fibrillar Aβ engagement of this receptor complex leads to the initiation of complex signaling events leading to protein tyrosine phosphorylation and activation of the src-family kinases Lyn and Fyn as well as the tyrosine kinase Syk [30,32,52]. Activation of these signaling cascades are linked to the synthesis and secretion of proinflammatory molecules and cytokines [28,30,52-58].

Microglia exposed to fAβ exhibit a respiratory burst leading to the release of superoxide anion [28,30,32,52,59]. Release of ROS is mediated through the fAβ cell surface receptor complex [31,32]. Furthermore, global inhibition of src-family tyrosine kinases or inhibition of phosphatidylinositol-3 kinase (PI3K) attenuates ROS production. These findings suggest that these kinases are involved in upstream signaling cascades responsible for activating NADPH oxidase assembly in response to fAβ peptides [28,30].

In both human AD brain tissue [27] and fAβ-treated cultured monocytes and primary microglia [28], there is a translocation of both the p47^phox ^and p67^phox ^subunits from the cytosol to the membrane. Fibrillar Aβ-stimulation also results in a relative increase in mRNA transcripts for both p47^phox ^and gp91^phox ^and an increase in p47^phox ^protein expression in monocytes primed with INFγ [59]. Recently, we have demonstrated that the Rac GTPase is also activated and subsequently translocated from the cytosol to the membrane of THP-1 monocytes in a fAβ-dependent manner [31]. A detailed examination of the mechanisms subserving oxidase activation has revealed that upon fAβ stimulation, the Vav guanine nucleotide exchange factor (GEF) is a key modulator of NADPH oxidase assembly in monocytes and primary microglia (Figure 3). Vav is responsible for the exchange of GDP for GTP on the Rac GTPase. In order for Vav to exert its GEF activity, it must be tyrosine phosphorylated [60]. The tyrosine phosphorylation of Vav requires fibril engagement of the Aβ cell surface receptor complex as well as activation of tyrosine kinase cascades involving Lyn or Syk [31]. Importantly, genetic deletion of Vav from primary microglia resulted in severe attenuation of ROS production following fAβ treatment [31].

Confirmation of NADPH oxidase participation in Aβ-induced ROS production has been obtained utilizing cells obtained from patients with the rare recessive genetic disorder, chronic granulomatous disease (CGD). This disease is characterized by a mutation in the genes that encode one of the essential subunits of the NADPH oxidase: p22^phox^, p47^phox^, p67^phox ^or gp91^phox^. These defects render the cells unable to generate H_2 _O_2 _in response to any agonist of the oxidase. Bianca and colleagues showed that monocytes and neutrophils from CGD patients fail to produce ROS in response to fAβ peptides or to phorbol 12-myristate 13-acetate (PMA) [28]. Subsequent studies have substantiated that the NADPH oxidase is essential for Aβ-induced ROS production. Elegant *in vivo *data from Park and colleagues assessed ROS production in the neocortex using hydroethidine fluoromicrography [61]. Fibrillar Aβ superfused through a cranial window increased ROS production in the neocortex. This effect could be abolished with the addition of a peptide inhibitor to the gp91^phox ^subunit. These authors further demonstrated that ROS levels were increased in the Tg2576 mouse model of AD; however, no signs of ROS production were evident in a mouse model where the Tg2576 mouse lacked the gp91^phox ^gene.

Surprisingly, the contribution of ROS to fAβ-stimulated neurotoxicity has not been extensively examined. Previous studies have reported that proinflammatory molecules secreted from fAβ-stimulated microglia lead to neuronal apoptosis [52,62]. However, it has only recently been appreciated that in a microglia/APP-expressing neuroblastoma cell co-culture model inhibition of ROS activity with superoxide dismutase or catalase (ROS scavengers) resulted in decreased neuronal cell death [63]. These authors validated their findings using a NADPH oxidase-deficient (gp91^phox ^null macrophage cell line in a similar co-culture model. The oxidase-deficient cells were unable to kill the APP-expressing neuroblastoma cells. Taken together, these findings argue that the interaction of Aβ with microglia and the assembly of the active microglial NADPH oxidase maybe largely responsible for the oxidative damage observed in the AD brain.

## Astrocytes and the NADPH oxidase

Astrocytes are the most abundant glial cell type in the brain and greatly outnumber microglia [64]. Historically, astrocytes were believed to function solely as supporting cells; however, this dogma has recently undergone substantial re-evaluation. Astrocytes are also involved with synaptic efficacy, neurogenesis, gliogenesis and even inflammatory processes [65,66]. In the AD brain, it has been postulated that the abundance of astrocytes results in their extensive exposure to Aβ from the earliest stages of the disease. Indeed, reactive astrocytes are found adjacent to senile plaques. Plaque-associated astrocytes upregulate the expression of the chemokines MCP-1 and RANTES, which act as chemoattractants for microglia, These cells also release the proinflammatory cytokines TNFα, IL1β, as well as nitric oxide (NO) [67].

The existence of an astrocytic NADPH oxidase is controversial. Shimohama and colleagues examined microglia, neurons and astrocytes in culture for the presence of either p67^phox ^or p47^phox^, and these oxidase subunits were not detected in astrocytes [27]. Additionally, in a co-culture model including neuron-enriched, microglia-enriched, and astrocyte-enriched cultures, the addition of fAβ failed to stimulate the generation of ROS in all cultures except the microglia [68]. These data support the prevailing view that astrocytes are not a significant source of ROS. However, recent findings have challenged this hypothesis. Abramov et al. have reported that astrocytes express the full complement of NADPH oxidase subunits [69]. In primary astrocytes cultures, NADPH oxidase activation was induced by exposure to fAβ peptides and this response was abolished by the addition of the NADPH oxidase inhibitors diphenylene iodonium (DPI), apocynin, or 4-(2-aminoethyl)-benzene-sulphonylfluoride [29,70]. Currently, the receptors and signaling pathways responsible for fAβ-dependent NADPH oxidase complex formation in astrocytes remain unknown. Unlike the fAβ multireceptor cell surface complex described in microglia, astrocytes must initiate complex formation differently in response to fAβ as preincubation of cells with a CD36 blocking antibody had no effect on fAβ-induced ROS generation [70]. The fAβ induced ROS production in astrocytes is postulated to be a consequence of the loss of mitochondrial membrane potential and glutathione depletion; however, exactly how fAβ activates the astrocytic NADPH oxidase remains unclear [29,70].

Abramov and colleagues have examined a potential role for astrocytic NADPH oxidase-derived ROS in fAβ-dependent neuronal death. In an astrocyte/neuron co-culture model, fAβ treatment caused cell death in both cell types but to a much larger extent in neurons. This response was almost completely reversed by treatment with the NADPH oxidase inhibitor DPI [29,70]. Moreover, treatment with antioxidants and glutathione precursors also decreased the neurotoxicity [29,70]. However, it remains uncertain how the astrocytes promote neuronal cell death.

## Neurons and the NADPH oxidase

Neuronal NADPH oxidase activity has not been widely examined. The presence of phagocytic NADPH oxidase (NOX2) subunits within cultured cortical and sympathetic neurons has been reported [71,72]. It has also recently been appreciated that the NOX4 subunit is present in mouse brain in cortex, in hippocampal neurons, and in Purkinje cells [73]. Neuronal cells respond to toxic insults including ischemia [73], zinc overload [72], and nerve growth factor deprivation [71] by inducing the expression and translocation of NADPH oxidase subunits. In the central nervous system, Noh and Koh (2000) were able to demonstrate increased NADPH oxidase-derived (NOX2) ROS production in cortical cultures in response to zinc exposure [72]. In response to fAβ peptides, however, neurons fail to generate an NADPH oxidase derived respiratory burst [68]. These findings suggest that the presence of NADPH oxidase complex subunits within neurons may mediate signaling pathways that regulate some other aspect of cellular response [74].

## Role of the NADPH oxidase in peroxynitrite formation and oxidative damage

There is substantial evidence supporting the fAβ-induced production of superoxide from the NADPH oxidase in microglia. In addition, neurons, microglia and astrocytes are capable of generating substantial amounts of nitric oxide (NO) through the inducible nitric oxide synthase (iNOS) [75-77]. Fibrillar Aβ peptides stimulate the induction of iNOS and NO production through an NADPH-dependent oxidative deamination of L-arginine [56,75,78,79]. *In vitro *studies have demonstrated that monocytes, microglia, and astrocytes release NO in response to fAβ and this response is enhanced by the proinflammatory cytokines [56,75,80]. Microglia/neuron co-culture studies reveal that the NO released from Aβ-stimulated microglia is toxic to neurons, and this effect is exacerbated by the addition of INFγ [81]. NO production is necessary for neuronal cell death as inhibition of nitrite production with a NO synthase inhibitor attenuates NO-induced neurotoxicity [81,82].

Inflammation-induced activation of microglial NADPH oxidase and iNOS has been reported to act synergistically to kill neurons through the formation of peroxynitrite [82]. Peroxynitrite is a potent oxidant with biological reactivity similar to that of the hydroxyl radical [83]. NO and superoxide form peroxynitrite at nearly the diffusion controlled rate [*k *= 6.7 × 10^9 ^M^-1 ^s^-1^] [84]. Peroxynitrite promotes the tyrosine nitration and nitrosylation of cysteines within cellular proteins. The addition of nitrite to tyrosine residues is extremely detrimental as it leads to both protein and enzyme dysfunction and eventual death of cultured neurons [82,85-87]. Peroxynitrite can directly oxidize proteins [88], lipids [89] and DNA [90].

Peroxynitrite is postulated to be associated with AD pathogenesis [86,91-94]. Levels of both dityrosine and nitrotyrosine are elevated in brain regions specifically affected in AD [91]. Protein nitration is also increased in the hippocampus of AD patients compared to age-matched controls, and nitrotyrosine is evident in, but not restricted to, neurons containing neurofibrillary tangles [92]. Recent advances in proteomics have also identified specific proteins which are nitrated in the AD brain including β-actin, enolase, and L-lactate dehydrogenase [94].

The role microglia play in peroxynitrite-mediated neurotoxicity has only recently been described. Aβ-mediated neuronal apoptosis in vitro is likely a consequence of the microglial secretion of proinflammatory cytokines including TNFα [3,95]. Importantly, TNFα stimulates increased iNOS in neuronal cells [62,96,97]. Combs et al. (2001) demonstrated that conditioned media from Aβ-stimulated primary microglia produce a TNFα/iNOS-dependent neuronal apoptosis, which could be rescued with the addition of either a TNFα antibody or a iNOS-selective inhibitor [62]. Levels of both intracellular neuronal NO and nitrotyrosine, a marker or peroxynitrite activity, were elevated by the addition of Aβ-conditioned media. An iNOS selective inhibitor eliminated the levels of both molecules [62]. Similarly, the addition of a peroxynitrite decomposition catalyst (FeTM PyP) blocked the neurotoxicity of Aβ-stimulated microglia, and a superoxide dismutase (SOD) mimetic (MnTM PYP), which blocks peroxynitrite formation by scavenging superoxide radicals, also ameliorates neuronal cell death [98]. Taken together, these data suggest that Aβ-stimulated production of peroxynitrite plays an important role in the pathogenesis of oxidative damage in the AD brain.

Oxidative damage directly stemming from peroxynitrite formation has been examined in the AD brain. Immunocytochemical 4-hydroxynonenal (HNE) analysis demonstrated increased lipid peroxidation in AD brain tissue [99,100]. Using 8-hydroxyguanine (8-OHdG), a widely studied biomarker for DNA oxidative damage, several groups have also demonstrated increased nucleic acid damage in the AD brain [101,102]. However, a direct linkage between these oxidative events and the microglial NADPH oxidase has not been established.

## Antioxidant therapy and the NADPH oxidase

Considerable attention has been devoted to antioxidants as a potential therapeutic intervention in AD. These compounds reduce oxidative stress by scavenging free radicals. A large number of candidate antioxidants exist including the monophenolic compounds: tocopherol, 17β-estradiol, and 5-hydroxytryptamine (serotonin) and the polyphenolic compounds: flavonoids, stilbenes, and hydroquinones [103]. Among these potential antioxidants, α-tocopherol (vitamin E) has been the most widely studied for the treatment of AD.

While previous studies have not directly analyzed the relationship between vitamin E supplementation and fAβ-dependent activation of the NADPH oxidase, there is evidence suggesting vitamin E could potentially inhibit an Aβ-induced respiratory burst. First, microglial cells treated with vitamin E transition into a "resting" morphological phenotype and downregulation the expression of adhesion molecules [104]. Second, *in vitro *studies demonstrate that BV-2 murine microglia or primary microglial pre-incubated with vitamin E have an attenuated respiratory burst in response to PMA. These cells also exhibit defects in the phosphorylation and translocation of p47^phox ^[105] and p67^phox ^[106] to the membrane. Third, vitamin E treatment decreases NO production and induction of TNFα and IL-1α in lipopolysaccaride (LPS)-stimulated microglial cells [107]. Early vitamin E supplementation also decreases lipid peroxidation [108]. Finally, vitamin E imparts a neuroprotective effect on neuronal cells in microglia-neuron cocultures stimulated with LPS [107]. Taken together, these findings provide compelling *in vitro *evidence that vitamin E could act to inhibit the Aβ-stimulated microglia inflammatory response. Specifically, vitamin E could inhibit ROS and RNI production.

In a transgenic mouse model of AD, dietary vitamin E reduced the formation of Aβ_1–40 _and Aβ_1–42 _and also reduced Aβ plaque burden in the brains of young but not aged Tg2576 mice [108]. The efficacy of dietary vitamin E has also been studied in clinical trials with patients with moderately severe AD. Daily treatment with vitamin E slowed the progression of the disease, but did not improve cognitive scores over a two-year period [109]. Zandi and colleagues more recently analyzed the relationship between antioxidant supplements and AD risk, and concluded that use of vitamin E and vitamin C in combination reduced the prevalence and incidence of AD in an elderly population; however, the use of either of these vitamins alone had no effect [110]. Finally, in patients diagnosed with mild cognitive impairment (MCI), a transitional state between normal brain aging and AD, daily vitamin E supplementation failed to prolong the rate of progression to AD [111]. These studies suggest that early dietary supplementation with vitamin E in combination with additional antioxidants may impart a protective effect against AD onset; however, it remains unclear how the effects of antioxidant treatment are mediated. Little attention has been focused on how antioxidant therapy might affect the proinflammatory responses of microglia or astrocytes to Aβ peptides.

## Statin therapy and the NADPH oxidase

The rationale for statin therapy as a potential treatment for AD arose from several epidemiological studies, which reported that hypercholesterolemia in midlife could predict the later occurrence of AD [112,113]. As a therapeutic agent, the primary action of statins is to competitively inhibit 3-hydroxy-3-methylglutaryl coenzyme A (HMG-CoA) reductase the rate limiting enzyme in the cholesterol biosynthetic pathway. Statins, therefore, block the *de novo *synthesis of cholesterol and ultimately lead to reduced plasma cholesterol levels. In addition to their ability to lower low-density lipoproteins, statins exhibit additional pleiotropic effects including modulation of inflammatory processes. The anti-inflammatory actions of statins are believed to arise from their ability to prevent the synthesis of isoprenoid intermediates that are responsible for the posttranslational addition of lipid attachments on the c-terminus of a variety of proteins including small GTPases such as Rac. The protein isoprenylation of Rac is critical for Rac's subcellular localization, interactions with RhoGDI, anchoring to the plasma membrane and ultimately to the activation of inflammatory signaling pathways [114].

In vitro evidence suggests the pleiotropic effects of statin treatment suppresses the microglia proinflammatory response to Aβ fibrils. Statin pretreatment abolished the fAβ-stimulated production of ROS in monocytes [115]. In addition, treatment of monocytes and microglia with simvastatin has been shown to uncouple the interaction between Rac and its negative regulator Rho-GDI and also disrupts Rac's ability to interact with the plasma membrane [116]. Both of these interactions rely on the isoprenylation of Rac. Therefore, reduction in ROS is likely a consequence of statin inhibition of Rac prenylation resulting in the inability of Rac to interact effectively to form the NADPH oxidase complex or RhoGDI.

The use of statins as a potential treatment for AD has received substantial attention based on two epidemiological studies that showed an association between statin use and a reduced incidence of AD. These studies demonstrated a ~70% reduction in AD incidence among patient populations receiving statin treatment [117,118]. Despite these promising findings, the use of statins in prospective clinical trials has yielded unsatisfactory results, failing to dramatically improve cognitive function or reduce serum plasma concentrations of Aβ_40 _or Aβ_42 _[119,120]. Statins remain an important tool to delineate the proinflammatory effects of fibrillar Aβ peptides in vitro; however, the efficacy of statins as a treatment for AD remains to be defined by large well-controlled clinical trials currently underway.

## Conclusion

Alzheimer's disease is characterized by a variety of proinflammatory responses that act in concert to promote the progressive pathophysiology associated with this disease. The results summarized in this review suggest that ROS and iNOS released through NADPH-dependent mechanisms contribute to the extensive oxidative damage found in the brains of AD patients. A greater understanding of the intracellular signaling events that give rise to prolonged inflammatory responses may facilitate the discovery of therapeutic agents that can ameliorate the prognosis for AD patients. Indeed, clinical studies have addresses the use of antioxidant and statins as potential therapies for the treatment of AD, unfortunately these early studies have yielded limited results. However, a disruption of oxidative damage through the sustained inhibition of the NADPH oxidase could possibly lead to an attenuation of the neuronal cell loss induced by fibrillar Aβ peptides.

## Competing interests

The author(s) declare that they have no competing interests.

## Authors' contributions

The authors contributed equally to the writing and editing of this manuscript.
